# Mental Health Continuum—Short Form: Confirmatory Factor Analysis (CFA) of Competing Models with Adolescents from Portugal

**DOI:** 10.3390/ejihpe15040043

**Published:** 2025-03-23

**Authors:** Luís Loureiro, José Santos, Cândida Loureiro

**Affiliations:** Health Sciences Research Unit: Nursing (UICISA: E), Nursing School of Coimbra, 3046-851 Coimbra, Portugal; jcsantos@esenfc.pt (J.S.); candida@esenfc.pt (C.L.)

**Keywords:** mental health literacy, mental well-being, positive mental health, adolescent, confirmatory factor analysis, scale validation

## Abstract

The concept of positive mental health (PMH) and therefore mental well-being (MWB) have taken center stage over the last few decades. The Mental Health Continuum—Short Form (MHC-SF) is one of the most important tools for assessing MWB. This study aims to assess the psychometric properties and dimensionality of the Portuguese version of the MHC-SF by comparing three competing models. Methods: Between January and March of 2021, a survey was conducted with a convenience sample of 495 Portuguese adolescents aged 12 to 18 years, enrolled in grades 7 to 12. Descriptive statistics and bivariate statistical tests and measures associated with confirmatory factor analysis (CFA) were calculated. Results: The results of the CFA support the idea that the bifactor model fits the data better than the other competing models. The indices support unidimensionality, namely the explained common variance (ECV = 0.81), the Percentage of Uncontaminated Correlations (PUC = 0.69), and the omega hierarchical (ωH = 0.89), and point to the existence of a general MHC-SF factor. The scale showed high reliability (ω = 0.95) in the general factor. The MHC-SF has concurrent validity, correlating with other scales that assess aspects related to mental health and well-being. Conclusion: The results point to the adequacy of the bifactor model and suggest the existence of a general factor of PMH, confirming that the total score of the scale can be used.

## 1. Introduction

Adolescence is a critical period in an individual’s life, characterized by significant transitions and changes in physiological, cognitive, emotional, and social aspects that can affect mental health (MH) and well-being (WB) (L. M. J. Loureiro et al., 2015; Rosa, 2018; Reinhardt et al., 2020; Kennes et al., 2020; Morgado et al., 2021). It is also considered a particularly vulnerable period for the onset of MH issues (C. C. Luijten et al., 2019; Yousefi Afrashteh & Janjani, 2023). Globally, the estimated prevalence of mental disorders among individuals aged 10–19 years is approximately 20.0% (World Health Organization, 2024). These challenges have been accompanied by a new perspective on MH and a broader approach to understanding WB (Kennes et al., 2020).

Over the last few decades, MH has moved from being understood as the mere absence of mental illness (MI) (L. Loureiro, 2024) to being understood as “a state of WB in which the individual realizes their own abilities, can cope with the normal stresses of life, can work productively and fruitfully, and is able to make a contribution to their community” (World Health Organization, 2004). The inclusion of WB in this definition began to incorporate positive feelings and positive functioning as key factors for MH (Galderisi et al., 2015). The inclusion of mental well-being (MWB) can be seen as re-energizing the concept of positive mental health (PMH) in line with the work of Jahoda (1958), who focused on the eudaimonic aspects of WB, and Gurin et al. (1969), who focused more on the hedonic aspects of subjective WB (Gautam et al., 2024) that were later proposed by Keyes (2002, 2005a, 2006, 2014). Keyes added emotional well-being (EWB), psychological well-being (PWB), and social well-being (SWB) as dimensions of PMH.

This tripartite view of PMH is associated with the development of the Mental Health Continuum-Short Form (MHC-SF) (Keyes, 2006) and is reflected in the concept of MH present in the dual continua model of MH (Keyes, 2002).

MH is defined as “a syndrome of symptoms of hedonia and positive functioning, operationalized by measures of subjective WB—individuals’ perceptions and evaluations of their lives and the quality of their functioning in life” (Keyes, 2005a). It includes the presence of symptoms associated with positive functioning (SWB and PWB) and the presence of positive emotions (EWB). The dual continua model of MH has had implications for both MH promotion and clinical practice by incorporating knowledge about diseases (pathogenesis—preventive MH) and knowledge about health and WB (salutogenesis), indicating that the presence of MWB does not necessarily imply the absence of MI and vice versa (L. Loureiro, 2024).

As proposed by Keyes (2002), MH comprises two continua, one for MH and another for WB. Although related, these continua are distinct. The MH (and MI) continuum comprises the absence of signs of mental and psychiatric illness at one extreme and the presence of psychiatric disorders at the other extreme. The WB continuum comprises the individual being emotionally well and functioning positively (flourishing) at one extreme and the individual not functioning emotionally well and not functioning appropriately (languishing) at the other extreme.

Keyes (2002) thus identifies three states of MH: flourishing, languishing, and moderate MH. Moderate MH includes individuals who do not meet the criteria for flourishing and languishing states.

It is in this context that Keyes presented the MHC-SF (Keyes, 2005b; Keyes et al., 2008), which encompasses the tripartite structure of WB (EWB, SWB, and PWB). Over the last decade, a substantial body of evidence has supported the existence of these three interrelated factors (Keyes, 2005b; Keyes et al., 2008), as theoretically proposed (Keyes, 2002). These conclusions have also been evidenced in different contexts and populations, both clinical and non-clinical (Iasiello et al., 2022), including in studies conducted in Portugal (Matos et al., 2010; Monteiro et al., 2021; Carvalho et al., 2016).

However, more recently, the correlated three-factor structure of the MHC-SF has been questioned, and although a substantial number of studies confirm this structure (Iasiello et al., 2022), some authors even point out the need to compare different models and competing structures (Franken et al., 2018; Reinhardt et al., 2020), with an emphasis on the bifactor model (Echeverría et al., 2017; Jovanović, 2015; Monteiro et al., 2021). This model “hypothesizes a general factor, onto which all items load, and a series of orthogonal (uncorrelated) skill-specific grouping factors. The model is particularly valuable for evaluating the empirical plausibility of subscales and the practical impact of dimensionality assumptions on test scores” (Dunn & McCray, 2020).

**Figure 1:**
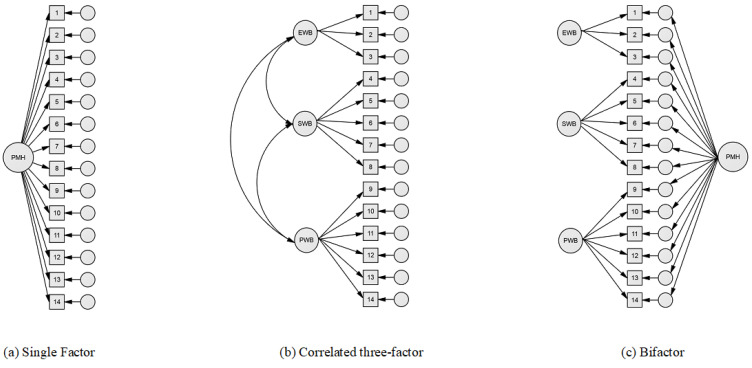
Models for confirmatory factor analysis evaluation.

In view of the above, particularly studies indicating inconsistencies in the structure and dimensionality of the MHC-SF, this study aims to evaluate the psychometric properties and dimensionality of the Portuguese version of the MHC-SF through confirmatory factor analysis (CFA) by comparing three competing models (Figure 1): single-factor, correlated three-factor, and bifactor models.

## 2. Materials and Methods

### 2.1. Participants

Between January and March of 2021, a survey was conducted with a convenience sample of 495 Portuguese adolescents, aged 12 to 18 years, enrolled in grades 7 to 12 in schools of a municipality in central Portugal. Data were collected in the classroom under the supervision of two researchers and the class teacher.

The mean age was 14.63 years (SD = 1.94), with 237 (47.9%) boys and 258 (52.15%) girls. In terms of the year of study, 317 (64.00%) attended 7th to 9th grade and 178 (36.00%) attended 10th to 12th grade. No statistically significant differences (t = −0.11; *p* > 0.05) were found when comparing age by gender. However, when comparing age by year of study, statistically significant differences (F = 583.89; *p* < 0.01) were found, which is to be expected, since higher educational levels imply older ages. In terms of year of study and gender, the differences were not statistically significant (χ^2^ = 0.30; *p* > 0.05).

### 2.2. Measures

Sociodemographic survey

This instrument consists of three questions about gender, age, and year of study.

#### 2.2.1. Mental Health Continuum—Short Form

The MHC-SF (Keyes et al., 2008) consists of 14 items that assess three dimensions of PMH. The questions are preceded by the following instructions: “Please answer the following questions about how you have been feeling during the past month. Place a check mark in the box that best represents how often you have experienced or felt the following”.

The EWB dimension concerns positive feelings and includes three items: 1. happy; 2. interested in life; and 3. satisfied with your life. The SWB dimension consists of five items: 4. that you have something important to contribute to society; 5. that you belong to a community (like a social group, your school, or your neighborhood); 6. that our society is becoming a better place for people like you; 7. that people are basically good; and 8. that the way our society works makes sense to you. The PWB dimension consists of six items: 9. that you like most parts of your personality; 10. are good at managing the responsibilities of your daily life; 11. that you have warm and trusting relationships with others; 12. that you have experiences that challenged you to grow and become a better person; 13. are confident to think or express your own ideas and opinions; and 14. that your life has a sense of direction or meaning to it. These last two dimensions relate to positive functioning. The items are rated on a Likert-type scale from 0 (never) to 5 (every day).

#### 2.2.2. Escala de Avaliação do Eu Resiliente (EAER, Resilient Self-Assessment Scale)

The EAER (Jardim & Pereira, 2006) includes 14 items rated on a Likert-type scale from 1 (never) to 5 (always). The scale consists of four dimensions: external support, inner strengths, social skills, and willingness to act. The scale showed high internal consistency (α total scale = 0.90). In this study, only the overall score of the scale was used.

#### 2.2.3. Multidimensional Life Satisfaction Scale for Adolescents (MLSSA)

The MLSSA (Segabinazi et al., 2010) consists of 52 items divided into seven components: Family, Self, Compared Self, School, Non-violence, Self-efficacy, and Friendship. The items are rated on a Likert-type scale from 1 (not at all) to 5 (very much). The scale showed high internal consistency (α total scale = 0.96). As with the EAER, only the overall scores of the scale were used.

### 2.3. Ethical Approval

Both this study and the survey were approved by the Directorate-General for Education through the Monitoring of School Surveys (MIME-DGE; Process no. 0224900009) and by the Ethics Committee of the Health Sciences Research Unit: Nursing (UICISA: E; P-736; P-738; P-739, P-740, P-741).

The following inclusion criteria were applied: agreeing to participate voluntarily in this study and signed parental or guardian consent, where required. The exclusion criteria were a diagnosis of MI and significant cognitive impairment that made it impossible to complete the questionnaires.

Given the characteristics of the sample (age < 18 years), the surveys were accompanied by a consent form to be signed by parents/guardians. In cases where the adolescents were 18 years of age, a consent form was provided on their behalf.

### 2.4. Data Analysis

This study used AMOS software (V. 27; SPSS Inc. Chicago, IL, USA), IBM-SPSS software (V. 28), and the Bifactor Indices Calculator tool (Dueber, 2017).

The appropriate summary statistics were calculated (means; standard deviations, skewness, kurtosis), as well as absolute and percentage frequencies. Student’s *t*-tests for independent groups, one-way ANOVA, the chi-squared test for two-way tables, and Pearson’s correlation coefficient and respective significance test were used.

Confirmatory factor analysis

The existence of outliers was assessed using the square of the Mahalanobis distance (D2). The normality of the variables was assessed using the skewness (Sk) and kurtosis (Ku) coefficients, in univariate and multivariate terms. Sk < 3 and Ku < 10 were used as reference values to meet the assumption of normality (Marôco, 2021).

The fit of the CFA was assessed using the following indices: χ^2^/df, the Comparative Fit Index (CFI), the Goodness of Fit Index (GFI), the Root Mean Square Error of Approximation (RMSEA), and the Akaike Information Criterion (AIC) (Kline, 2023).

The fit of the models was determined using the factor loadings and the individual reliability of the items. Model fit was achieved using the modification indices (>11.0 and *p* < 0.001).

In the bifactor model, the explained common variance (ECV), the H index (H), the Factor Determinacy (FD), and the Percentage of Uncontaminated Correlations (PUC) were also calculated.

The Omega (ω), omega hierarchical (ωH), and Relative Omega (ωR) were used to assess reliability (Hair et al., 2010; Liu et al., 2023).

## 3. Results

Table 1 shows the descriptive statistics for the variables. The Sk and Ku values of the individual items did not diverge from the values expected in a normal distribution (univariate and multivariate), given that [|Sk| < 3.00] and [|Ku| < 10.00] (Marôco, 2021).

In order to respond to the main objective of this study, the next step was to assess model fit. In this case, the single-factor, three-factor, and bifactor models were analyzed using the fit indices shown in Table 2.

As can be seen, the single-factor model had the least satisfactory fit. In terms of χ^2^/df, the value obtained (6.96) indicates a poor fit (>0.5). These values are in line with those obtained in other indices such as CFI (0.88) and GFI (0.84), which indicate a poor fit [0.80; =0.90]. The RMSEA value was 0.11 [RMSEA > 0.10], indicating an unacceptable fit.

With regard to the correlated three-factor model, the values of most indices improved compared to the single-factor model. Although the χ^2^/df (3.44) indicates a poor fit, the CFI (0.88) and GFI (0.93) indicate a good fit. The RMSEA value (0.07) indicates a good fit (reference values: [0.05; 0.10]). The AIC value indicates that the three-factor model (AIC = 316.86) has a better fit than the single-factor model (603.73).

Finally, the model with the best fit indices is the bifactor model, although the χ^2^/df (2.24) exceeds the critical threshold for a good fit (1.00 < (χ^2^/df) < 2.00). The other indices point to an optimum fit, namely CFI = 0.98, GFI = 0.96, and RMSEA = 0.05. A comparison of the three AIC measures indicates that the bifactor model has the best fit (AIC = 225.10).

A different approach was used to validate the conclusions on the two models with the best fit indices. The Chi-square values obtained from the models with the best fit indices were compared (Marôco, 2021): the correlated three-factor model (χ^2^(Three-factor) = 254.86) and the bifactor model (χ^2^(bifactor) = 113.76). Thus, the following is true:

χ^2^dif = χ^2^(Three-factor) − χ^2^(bifactor) and df = df(Three-factor) − df = df(bifactor)

χ^2^dif = 254.86 − 141.10 = 113.76 and df = 74 − 63 = 11

In this case, the test statistic will be χ^2^dif = 113.76 and df = 11. The Chi-square distribution (Marôco, 2021), for alpha of 0.05 and df = 11, indicates that the theoretical Chi-square value will be 19.68.

As a result, and as emerged from the initial comparison of indices, the results indicate and confirm that the bifactor model is significantly better than the correlated three-factor model.

Table 3 shows the factor loadings for the three structures under analysis. It should be noted that, with regard to the correlated three-factor model, the correlation values between the three latent factors were r = 0.78 (*p* < 0.01) between EWB and SWB, r = 0.85 (*p* < 0.01) between EWB and PWB, and r = 0.82 (*p* < 0.01) between SWB and PWB.

Table 3 shows that the factor loadings of the general factor in the bifactor model are similar to those of the single-factor model, and the factor loadings of the dimensions in the bifactor model are comparatively smaller than the factor loadings of the correlated three-factor model, which suggests a high influence of the general factor in the bifactor model, with the dimensions having less influence on the variance of the items.

The factor loadings of the general factor in the bifactor model are all statistically significant (*p* < 0.001), ranging from 0.61 to 0.79, similar to those observed in the single-factor model. The correlated three-factor model also shows that all factor loadings are statistically significant (*p* < 0.001).

The analysis of the factor loadings of the bifactor model, specifically in the three specific domains, revealed that some of the values are lower than 0.30, the minimum acceptable value (Hair et al., 2010), but 12 of these items are statistically significant (*p* < 0.001). Only two items were not statistically significant, namely item 5 (that you belong to a community (like a social group, your school, or your neighborhood) and item 11 (that you have warm and trusting relationships with others), which may indicate that the general factor explains a high percentage of the variance in the items.

The following indices were calculated for the bifactor model (Dueber, 2017): ECV and PUC. Both measures are used to assess the degree of unidimensionality (Liu et al., 2023). In this case, the values obtained were ECV = 0.81 and PUC = 0.69 (close to 0.70), which means that the general factor explains a high percentage of the variance in the items (approximately 81%). It is common to assume that when the ECV and PUC measures are both >0.70, the relative bias will be slight, and the common variance can essentially be considered unidimensional (Rodriguez et al., 2016). If ECV is >0.80, the relative bias tends to be <5.0%.

With regard to the FD and H index, the values for the general factor are 0.95 and 0.93. These values are considered good, but they are not found in the dimensions, which suggests that the MHC-SF has a strong general factor (Dueber, 2017). An H index value > 0.80 suggests a well-defined latent variable.

In terms of reliability, the ω, ωH, and ωR were used. The value obtained in the general factor was ω = 0.95, ω = 0.88 in the EWB dimension, ω = 0.89 in the SWB dimension, and ω = 0.87 in the PWB dimension. All these values point to high reliability, which is higher in the general factor.

The Cronbach’s alpha values for the single factor (α = 0.93) and for each of the three factors (α = 0.84 for EWB, α = 0.87 for SWB, and α = 0.86 for PWB) indicate high internal consistency, supporting the reliability of the MHC-SF.

When the general factor was controlled for, variance decreased in the MHC subscales. The ωH ranged from ωH = 0.07 in the PWB dimensions and ωH = 0.19 in the EWB and SWB dimensions, while it remained high in the general factor (ωH = 0.89). The ωR indicates that only 23.0% of the variance in the EWB factor, 21.0% in the SWB factor, and 8.0% in the PWB factor is independent of the general factor.

Convergent validity was also analyzed by correlating the total score of the MHC-SF with the total global scores of the EAER and MLSSA. The results indicate a positive, strong, linear, and statistically significant correlation between the EAER and the overall MHC-SF score (r = 0.78; r^2^ = 0.61; *p* < 0.001), as well as a strong, positive, linear, and statistically significant correlation between MLSSA and the MHC-SF global score (r = 0.79; r^2^ = 0.62; *p* < 0.001).

Finally, the coding method proposed by Keyes et al. (2008) was used to create categorical diagnoses, placing the adolescents in one of the three states of languishing, flourishing, or moderate MH.

In this sample of adolescents, the results indicate that 4.2% are languishing, 36.6% are moderately mentally healthy, and 59.2% are flourishing.

When the categorical diagnoses were compared according to gender (Table 4), the chi-square test revealed a statistically significant association between the variables (χ^2^_(2)_ = 20.28; *p* < 0.001; Kramer V = 0.20). The results also show that more girls than expected are in the languishing and moderate MH states and that boys tend to be more in the flourishing state than girls.

## 4. Discussion

This study aimed to test three competing models (single-factor, correlated three-factor, and bifactor) in relation to the structure of MWB measured by the MHC-SF.

The results indicate that the bifactor model presents a better fit to the data than the competing models, which is consistent with other studies carried out with the instrument in samples from different social, economic, and cultural backgrounds, including clinical and non-clinical samples (de Bruin & du Plessis, 2015; Jovanović, 2015; Karaś et al., 2014; C. C. Luijten et al., 2019; Fonte et al., 2020; Reinhardt et al., 2020; Kennes et al., 2020; Hernández-Torrano et al., 2021; Monteiro et al., 2021; C. Luijten, 2022; Piqueras et al., 2022; Yeo & Suárez, 2022; Yousefi Afrashteh & Janjani, 2023; Söderqvist & Larm, 2023). Even so, a substantial number of studies point to the correlated three-factor structure model (Carvalho et al., 2016; Karaś et al., 2014).

The results found in this study suggest the existence of a general factor (PMH) and support the calculation of a total MHC-SF score, so the calculation of scores for the three independent subscales may be questionable.

In the correlated three-factor model, the results found in this study show positive and strong correlations between the three subscales, which is also observed in other studies (Carvalho et al., 2016; Matos et al., 2010; de Bruin & du Plessis, 2015; Jovanović, 2015; Karaś et al., 2014; C. C. Luijten et al., 2019; Fonte et al., 2020; Reinhardt et al., 2020; Kennes et al., 2020; Hernández-Torrano et al., 2021; Monteiro et al., 2021; C. Luijten, 2022; Piqueras et al., 2022; Yeo & Suárez, 2022; Yousefi Afrashteh & Janjani, 2023; Söderqvist & Larm, 2023). However, the fit indices always indicate a better fit of the bifactor model.

With regard to reliability, the omega indices had high values for the general factor (ω = 0.95) and the three latent factors (ranging from 0.87 to 0.89). However, when the variance associated with the general factor was controlled for, the omega values for the subscales were low (ranging from 0.07 to 0.19), below recommended (ωH = 0.75), or the minimum acceptable value (ωH = 0.50) (Reise et al., 2013). These results for the ωH show that it is not possible to unreservedly accept the subscales as separate dimensions of PMH, which may justify not using the subscales.

As mentioned in the Results Section, although the factor loadings of the single-factor model and the general factor of the bifactor model are very identical, the comparison between the factor loadings of the three subscales of the three-factor model and the separate factors of the bifactor model shows that the difference is high, which is also found in other studies (Echeverría et al., 2017; Monteiro et al., 2021). In addition, the results obtained in the ECV show that the general factor explains a high percentage of the common variance extracted (81.0%) compared to the factors, whose values range from 0.10 to 0.26. Similar results have been found in other studies (Jovanović, 2015; Monteiro et al., 2021), suggesting that the MHC-SF is primarily a unidimensional measure. However, it should be noted, for example, that factors have different values, with the SWB factor showing an ECV = 0.26, an H = 0.53, and ωH = 0.19. These values differ, for example, from the PWB subscale, which shows comparatively lower values in these measures. Therefore, further studies should be carried out to assess and clarify the existence of a general factor that overlaps with the WB dimensions.

Finally, the results of the correlations between the total score of the MHC-SF and the global scores of the EAER and MLSSA confirm convergent validity because both correlations indicate that better PMH is associated with improved resilience, in the same way that better MH is associated with greater life satisfaction in adolescents and young people.

The results obtained in relation to the PMH categories are consistent with another study carried out in a Portuguese context with samples of adolescents (Matos et al., 2010). The authors found that most adolescents (44.7%) were flourishing, 38.9% had moderate MH, and 16.4% were languishing. As this study (Matos et al., 2010) dates from 2010, there has been an improvement in the MH of adolescents, namely, a decrease in the percentage of adolescents languishing, from 16.4% to 4.2%, an increase in those flourishing, from 44.7% to 59.2%, and a slight decrease in those with moderate MH, decreasing by only 2.3% (38.9–36.6).

It should be noted that the pattern regarding the percentages of PMH states in this study was similar for both girls and boys.

With regard to the differences between the different states of PMH and gender, the results are in line with those obtained by Matos et al. (2010). The percentage of adolescents in the languishing and moderate MH states is higher in girls than in boys, whereas the percentage of adolescents in the flourishing state is higher in boys than in girls.

These results, combined with recent documents referring to the Portuguese population (Conselho Nacional de Saúde, 2019), point to the need to invest in the mental health literacy (MHL) of adolescents and young people, emphasizing PMH as one of the dimensions of the MHL concept (L. Loureiro, 2024). In this context, the MHC-SF is a valuable tool for assessing PMH.

## 5. Conclusions

The MHC-SF is a valid and reliable instrument for assessing PMH. Although the data suggest the existence of a general factor of PMH, they do not provide sufficient statistical support in this study to recommend without reservation the scores of the subscales that assess the different dimensions of WB. Future studies should include larger samples and evaluate other models, such as the second-order model.

Evaluating other statistical models, such as the second-order model, allows researchers to better represent the complexity of the construct by capturing hierarchical structures and understanding the relationship between the general factor and specific factors. The second-order model can improve model fit by providing a more flexible and accurate representation of the data. It should be noted that studies conducted with different populations may yield different results than those found in this study.

Therefore, it can be concluded that the fit indices show that the bifactor model has a better fit than the competing models tested.

## Figures and Tables

**Table 1:** Descriptive statistics of MHC-SF items, factors, and total (N = 495).

Items	Minimum	Maximum	*M*	*SD*	*Sk*	*Ku*
MCH1 (EWB)	1.00	5.00	3.83	0.79	−0.96	1.76
MCH2 (EWB)	1.00	5.00	4.05	1.11	−1.13	0.56
MCH3 (EWB)	1.00	5.00	3.70	1.19	−0.86	−0.07
MCH4 (SWB)	1.00	5.00	3.14	1.34	−0.38	−1.06
MCH5 (SWB)	1.00	5.00	3.56	1.39	−0.68	−0.81
MCH6 (SWB)	1.00	5.00	3.03	1.38	−0.22	−1.23
MCH7 (SWB)	1.00	5.00	3.11	1.29	−0.36	−1.01
MCH8 (SWB)	1.00	5.00	2.92	1.35	−0.12	−1.25
MCH9 (PWB)	1.00	5.00	3.62	1.30	−0.79	−0.47
MCH10 (PWB)	1.00	5.00	3.58	1.20	−0.81	−0.19
MCH11 (PWB)	1.00	5.00	3.89	1.15	−1.1	0.58
MCH12 (PWB)	1.00	5.00	3.68	1.25	−0.83	−0.25
MCH13 (PWB)	1.00	5.00	3.40	1.26	−0.58	−0.63
MCH14 (PWB)	1.00	5.00	3.69	1.35	−0.78	−0.61
Multivariate						66.86
EWB	3.00	15.00	11.60	2.76	−10.55	5.14
SWB	3.00	25.00	15.48	5.97	−5.18	−2.32
PWB	4.00	30.00	21.74	6.12	−9.55	3.59
Total MHC	6.00	68.00	48.77	13.47	−7.64	1.50

**Table 2:** Model fit statistics for the tested CFA models.

Models	χ^2^	*df*	χ^2^/*df*	CFI	RMSEA	AIC	GFI
Single-factor	547.73	77	6.96 ***	0.88	0.11	603.73	0.84
Three-factor	254.86	74	3.44 ***	0.96	0.07	316.86	0.93
Bifactor	141.10	63	2.24 ***	0.98	0.05	225.10	0.96

*** *p* < 0.001.

**Table 3:** Factor loadings for the single-factor, correlated three-factor, and bifactor models of MHC—SF.

Items	Single-Factor	Three-Factor			Bifactor			
	PMH	EWB	SWB	PWB	PMH	EWB	SWB	PWB
MCH1 (EWB)	0.64 ***	0.71 ***			0.61 ***	0.40 ***		
MCH2 (EWB)	0.76 ***	0.84 ***			0.76 ***	0.35 ***		
MCH3 (EWB)	0.78 ***	0.86 ***			0.77 ***	0.40 ***		
MCH4 (SWB)	0.77 ***		0.76 ***		0.76 ***		0.18 ***	
MCH5 (SWB)	0.62 ***		0.60 ***		0.63 ***		0.07	
MCH6 (SWB)	0.73 ***		0.84 ***		0.66 ***		0.61 ***	
MCH7 (SWB)	0.74 ***		0.81 ***		0.68 ***		0.45 ***	
MCH8 (SWB)	0.77 ***		0.85 ***		0.72 ***		0.46 ***	
MCH9 (PWB)	0.76 ***			0.80 ***	0.76 ***			0.22 ***
MCH10 (PWB)	0.65 ***			0.67 ***	0.65 ***			0.17 ***
MCH11 (PWB)	0.67 ***			0.67 ***	0.69 ***			0.02
MCH12 (PWB)	0.58 ***			0.61 ***	0.56 ***			0.25 ***
MCH13 (PWB)	0.67 ***			0.71 ***	0.66 ***			0.33 ***
MCH14 (PWB)	0.77 ***			0.81 ***	0.79 ***			0.23 ***
Explained Common Variance (ECV)					0.81	0.22	0.26	0.10
Factor Determinancy (FD)					0.95	0.68	0.81	0.53
H Index					0.93	0.34	0.53	0.25
Omega (ω)					0.95	0.88	0.89	0.87
Omega Hierarchical (ω_H_)					0.89	0.19	0.19	0.07
Relative Omega (R_ω_)					0.94	0.23	0.21	0.08
Cronbach’s Alpha (α)	0.93	0.84	0.87	0.86				

*** *p* < 0.001.

**Table 4:** Category diagnosis of PMH by gender.

Category Diagnosis of PMH	Gender		Total
	Male	Female	
Languishing	5 (23.8)	16 (76.2)	21 (4.2)
Moderate	68 (37.6)	113 (62.4)	181 (36.6)
Flourishing	164 (56.0)	129 (44.0)	293 (59.2)
Total	237 (47.9)	258 (52.1)	495

## Data Availability

The datasets generated and analyzed during the current study are available from the corresponding author upon reasonable request. Requests will be reviewed and granted in compliance with ethical and legal considerations.

## Abbreviations

The following abbreviations are used in this manuscript:

AIC	Akaike information criterion
CFA	Confirmatory factor analysis
CFI	Comparative fit index
Df	Degree of freedom
EAER	Escala de Avaliação do Eu Resiliente
ECV	Explained common variance
EWB	Emotional well-being
FD	Factor Determinancy
GFI	Goodness of Fit index
H	H index
Ku	Kurtosis
M	Mean
MH	Mental Health
MHC	Mental Health Continuum
MI	Mental Illness
MLSSA	Multidimensional Life Satisfaction Scale for Adolescents
PMH	Positive mental health
PUC	Percent of Uncontaminated Correlations
PWB	Psychological well-being
RMSEA	Root Mean Square Error of Approximation
SD	Standard deviation
Sk	Skewness
SWB	Social well-being
WB	Well-being
